# Rapid fluorescent reporter quantification by leaf disc analysis and its application in plant-virus studies

**DOI:** 10.1186/1746-4811-10-22

**Published:** 2014-07-05

**Authors:** Fabio Pasin, Satish Kulasekaran, Paolo Natale, Carmen Simón-Mateo, Juan Antonio García

**Affiliations:** 1 Centro Nacional de Biotecnología (CNB-CSIC), Darwin 3, Madrid 28049, Spain

**Keywords:** Fluorescent protein, Fluorescence spectroscopy, Microtiter plate, RNA silencing, Plant virus

## Abstract

**Background:**

Fluorescent proteins are extraordinary tools for biology studies due to their versatility; they are used extensively to improve comprehension of plant-microbe interactions. The viral infection process can easily be tracked and imaged in a plant with fluorescent protein-tagged viruses. In plants, fluorescent protein genes are among the most commonly used reporters in transient RNA silencing and heterologous protein expression assays. Fluorescence intensity is used to quantify fluorescent protein accumulation by image analysis or spectroscopy of protein extracts; however, these methods might not be suitable for medium- to large-scale comparisons.

**Results:**

We report that laser scanners, used routinely in proteomic studies, are suitable for quantitative imaging of plant leaves that express different fluorescent protein pairs. We developed a microtiter plate fluorescence spectroscopy method for direct quantitative comparison of fluorescent protein accumulation in intact leaf discs. We used this technique to measure a fluorescent reporter in a transient RNA silencing suppression assay, and also to monitor early amplification dynamics of a fluorescent protein-labeled potyvirus.

**Conclusions:**

Laser scanners allow dual-color fluorescence imaging of leaf samples, which might not be acquired in standard stereomicroscope devices. Fluorescence microtiter plate analysis of intact leaf discs can be used for rapid, accurate quantitative comparison of fluorescent protein accumulation.

## Background

Reporter genes and their products are valuable tools for plant studies, due to the ease of imaging and quantification of the proteins encoded [1]. Fluorescent proteins are widely employed as reporters, since they have no requirements for exogenous substrate/co-factors and do not interfere with cell growth or function [2]. These proteins can be detected and imaged in live tissue without cell lysis or biochemical analysis, and they allow optical exploration of cell structures and molecule dynamics as well as pathogen monitoring with minimal sample preparation [3].

Use of fluorescent protein as a quantitative reporter includes evaluation of new vectors for heterologous protein expression and of promoter activity, translational regulation and transient RNA silencing [4-8]. In plant pathology and symbiosis studies, fluorescent proteins are an important aid for monitoring infection/colonization onset and spreading, and thus facilitate comprehension of host-microbe interactions. Since the first demonstrations that plant viruses are useful vectors for foreign sequence transfer to their hosts [9-12], several genes were shown to be suitable RNA virus reporters; they include those that encode chloramphenicol acetyltransferase, firefly and *Renilla* luciferases, β-glucuronidase, anthocyanin biosynthesis transcription factors, and *Aequorea victoria* green fluorescent protein (GFP) [12-18].

Compared to other markers, fluorescent protein genes inserted into viral genomes offer good reporter stability [19], viral localization to individual cells, and monitoring of co-infection with differently-labeled viruses [20,21]. A further advantage of these proteins is that their fluorescence intensity is directly proportional to protein amount and can be used for quantification [22,23]. Although GFP fluorescence can be quantified by image analysis [24,25], this involves time-consuming steps that can be overcome by spectrofluorometric measurement of intact plant organs or protein extracts from GFP-expressing samples [23,26,27].

A microplate assay was recently described that measures luciferase activity in intact leaf discs [28]. In a similar approach, here we evaluated the use of 96-well plate readers for rapid quantification of two *A. victoria* GFP variants, the ultraviolet (UV)-excitable mGFP5 [29] and a mutant with enhanced brightness sGFP(S65T) [30]. The method was applied in viral RNA silencing suppressor studies and in accumulation monitoring of GFP-labeled *Plum pox virus* (PPV) clones. A palette of engineered monomeric fluorescent proteins was expressed transiently in plants (Table 1) and shown to be easily quantifiable by direct leaf disc analysis.

**Table 1:** Reporter proteins and fluorescence analysis conditions evaluated

**Reporter**	**Laser scanner imaging**		**Plate reader FI quantification**		**Species**	**Structure**	**Ref.**
	**Laser (nm)**	**Em (nm)**	**Ex (nm)**	**Em (nm)**			
mTagBFP2	n.a.^1^	n.a.	400/9	455/20	*Entacmaea quadricolor*	Monomer	[31]
mTFP1	457	526SP	450/9	480/20	*Clavularia sp.*	Monomer	[32]
mGFP5	488	526SP	485/9	535/20	*Aequorea victoria*	Weak dimer	[29]
sGFP(S65T)	488	526SP	485/9	535/20	*Aequorea victoria*	Weak dimer	[30]
mNeonGreen	n.t.^2^	n.t.	500/9	530/20	*Branchiostoma lanceolatum*	Monomer	[33]
mPapaya1	532	555/20	520/9	550/20	*Zoanthus sp.*	Monomer	[34]
TagRFP-T	532	580/30	560/9	595/20	*Entacmaea quadricolor*	Monomer	[35]

^1^n.a., not applicable.

^2^n.t., not tested.

## Results and discussion

### Laser scanner imaging of *Nicotiana benthamiana* leaves

**Figure 1:**
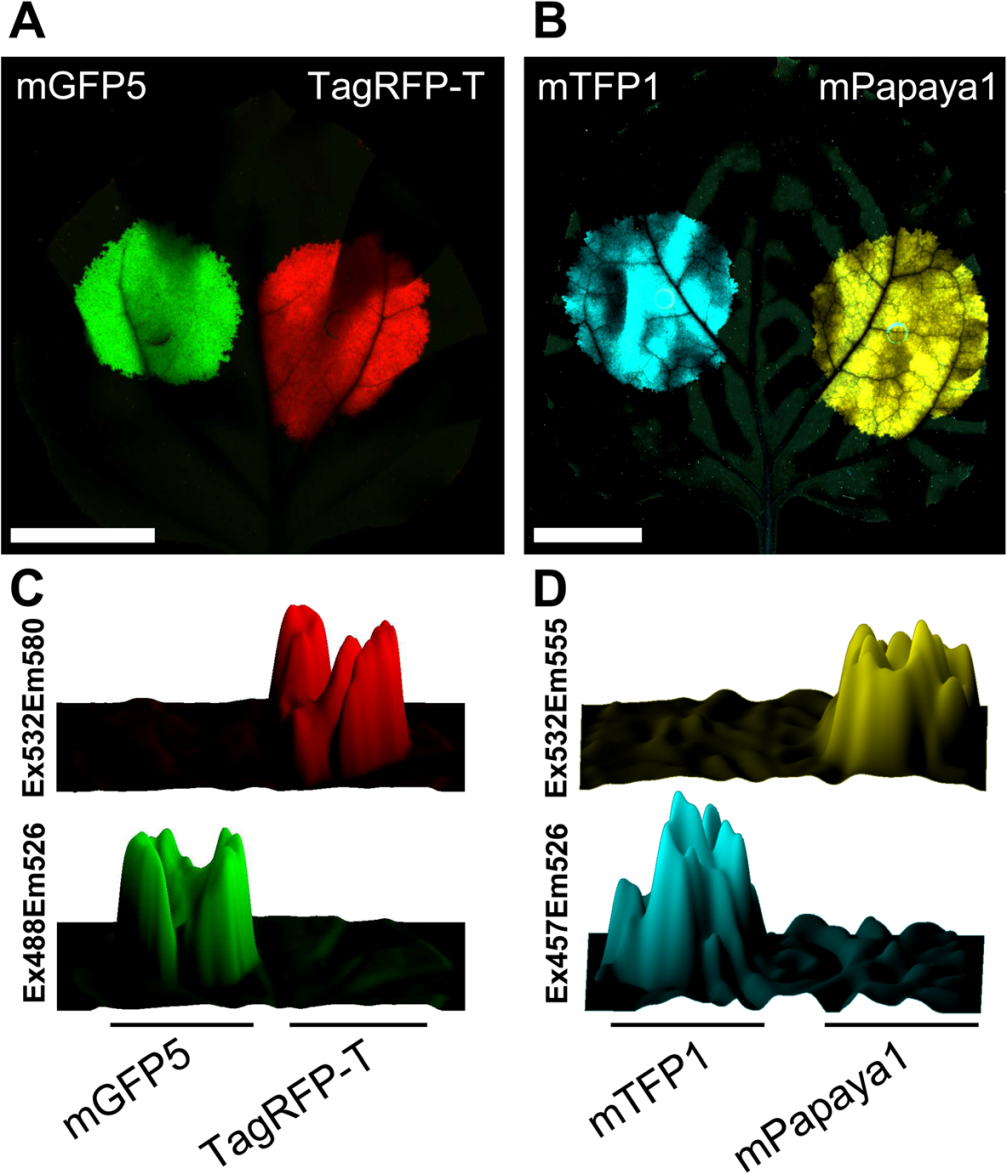
**Laser scanner imaging of fluorescent protein-expressing leaves.** Fluorescent proteins were transiently expressed by co-infiltrating *N. benthamiana* leaf tissue with an *Agrobacterium* pSN.5 p19 culture plus cultures of *Agrobacterium* containing pBin-35S-mGFP5 (mGFP5), pSN.5 TagRFP-T (TagRFP-T), pSN.5 mTFP1 (mTFP1) or pSN.5 mPapaya1 (mPapaya1). Fluorescence was imaged by leaf laser scanning. **(A)** Signal acquired at 6 dpa for TagRFP-T (red) and mGFP5 (green); green and red channel images were merged. Scale bar, 2 cm. **(B)** Signal at 3 dpa for mTFP1 (cyan) and mPapaya1 (yellow); cyan and yellow channel images were merged. Scale bar, 2 cm. **(C,D)** Surface plots of infiltrated patches from above images.

GFP variants such as mGFP5 [29], which can be excited by long-wavelength ultraviolet (UV) light, are used frequently in plant studies of species other than the small-sized *Arabidopsis*, since fluorescence imaging of whole specimens is constrained by objective lens size of fluorescence (stereo)microscopes. The need for fluorescence microscopes is overcome by use of UV lamps as excitation sources, although this restricts fluorophore choice and limits multi-fluorescence imaging. Scanners with excitation lasers at 457, 488, 532, and 633 nm are used for fluorescence imaging in two-dimensional difference gel analysis systems [36] and have a relatively large glass platen (for example, 35 cm × 43 cm, in the Typhoon 9400). As a 633 nm laser might be unsuited to leaf tissue imaging due to interference from chlorophyll autofluorescence [37], we tested whether 457, 488 and 532 nm lasers can be used for imaging *N. benthamiana* leaves that transiently express fluorescent proteins. Plant expression vectors bearing coding sequences for mGFP5 or a monomeric red fluorescent protein TagRFP-T [35] were delivered to plants by *Agrobacterium* infiltration. *Tomato bushy stunt virus* p19 RNA silencing suppressor was co-expressed to increase yield of the heterologous proteins delivered [38]. At 6 days post-agro-infiltration (dpa), *N. benthamiana* leaf fluorescence was acquired after excitation with 488 nm and 532 nm lasers. A strong signal was detected in leaf patches expressing the fluorescent proteins. Only background signal was detected in non-infiltrated leaf areas and when non-optimal excitation/emission conditions were used, i.e., mGFP5-expressing patches imaged with TagRFP-T settings (Ex532/Em580) and TagRFP-T-expressing patches imaged with mGFP5 settings (Ex488/Em526) (Figure 1A, C). To expand fluorophore choice, we tested a cyan (mTFP1; [32]) and a yellow (mPapaya1; [34]) fluorescent protein, and found them to be easily imaged in agro-infiltrated leaves (Figure 1B, D). These results support the suitability of mGFP5/TagRFP-T and mTFP1/mPapaya1 pairs for laser scanner bicolor imaging in plants.

### Spectral properties and quantification of plant-expressed fluorescent proteins

**Figure 2:**
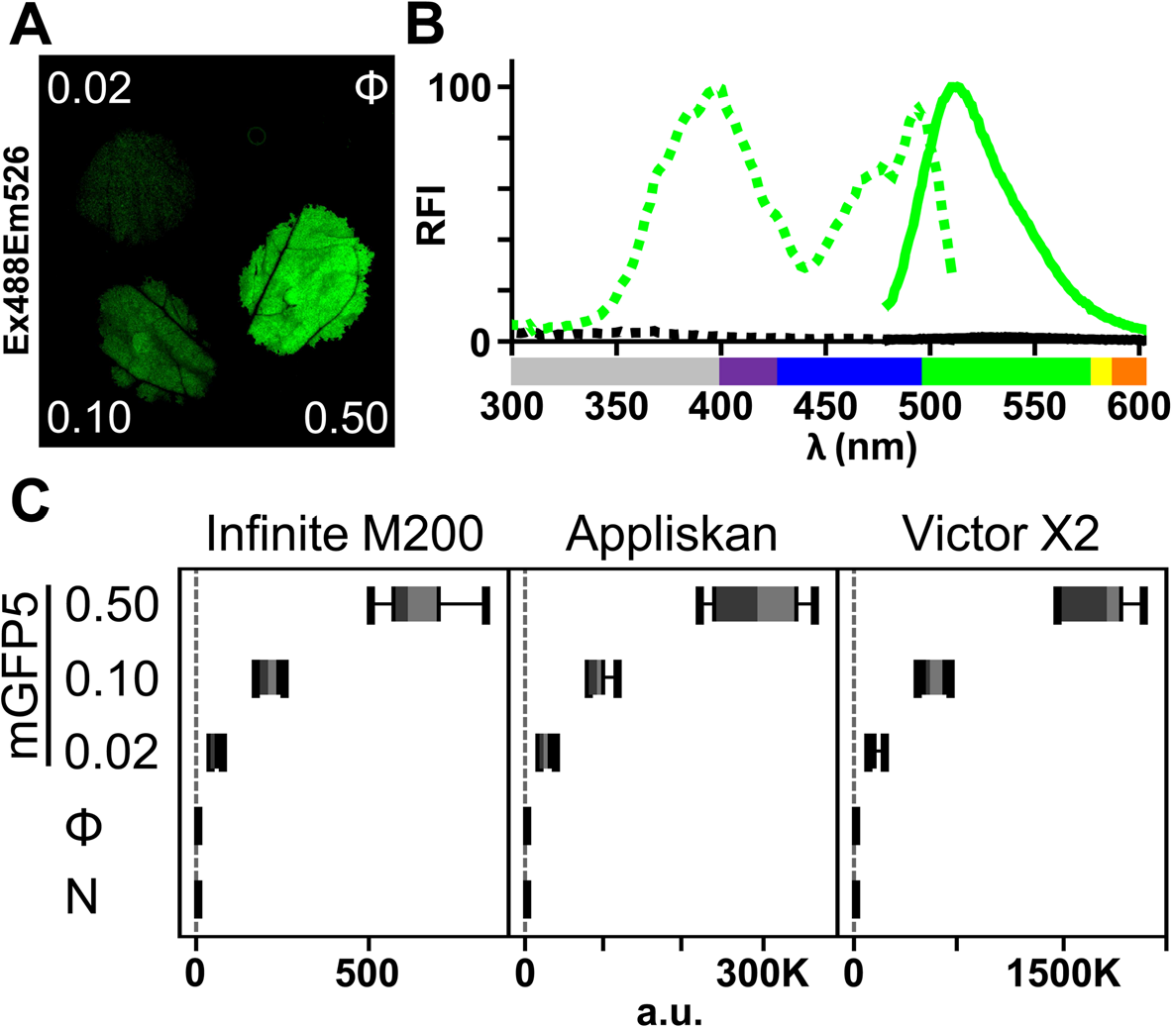
**Spectral properties and fluorescence quantification of GFP.** Fluorescent protein was transiently expressed by co-infiltrating *N. benthamiana* leaf tissue with an *Agrobacterium* pSN.5 p19 culture plus a strain with no expression vector (Φ), or 5-fold dilutions of *Agrobacterium* containing pBin-35S-mGFP5 (mGFP5 at OD_600_ 0.50, 0.10 and 0.02). **(A)** At 3 dpa, mGFP5 (green) fluorescence was imaged by leaf laser scanning. **(B)** In a plate reader, excitation (dotted lines) and emission spectra (solid lines) were measured from leaf discs of tissue agro-infiltrated with mGFP5 (green) or no expression vector (black). Relative fluorescence intensity (RFI) was plotted using mGFP5 peaks equal to 100. Ultraviolet (UV) wavelengths are in gray, visible spectrum colors were assigned as described [39]. **(C)** Box-plot graphs show quantification values from *n* = 8 samples/condition. Fluorescence intensity of leaf discs agro-infiltrated with mGFP5 strain dilutions, no expression vector (Φ) or non-treated samples (N) was acquired in monochromator-based (Infinite M200) and two filter-based (Appliskan and Victor X2) plate readers. Fluorescence intensity is expressed in arbitrary units (a.u.).

A fluorescence signal acquired by laser scanner imaging is suitable for quantitative comparisons (Figure 1C, D), as is done routinely in proteomic studies [36]. Image analysis can be a lengthy process, however, and signal quantification can be affected if leaf lamina occupy different focal planes during the acquisition step. As microtiter plate readers are available for medium-high throughput analysis, we used a monochromator-based plate reader to analyze the fluorescence signal from intact leaf discs collected from agro-infiltrated patches (Figure 2A). We found that fluorescence properties of mGFP5 could be measured without extract preparation (Figure 2B), and excitation and emission spectra closely resembled those reported [29]. Five-fold dilutions of the mGFP5-*Agrobacterium* strain were used in a transient expression assay. Fluorescence intensity values were consistent with the amount of bacteria delivered (Pearson R^2^ = 0.9855; *n* = 4; Infinite M200 values were considered) and independent of the fluorescent plate reader used (Figure 2C).

### Rapid fluorometer GFP quantification in transient RNA silencing assays

**Figure 3:**
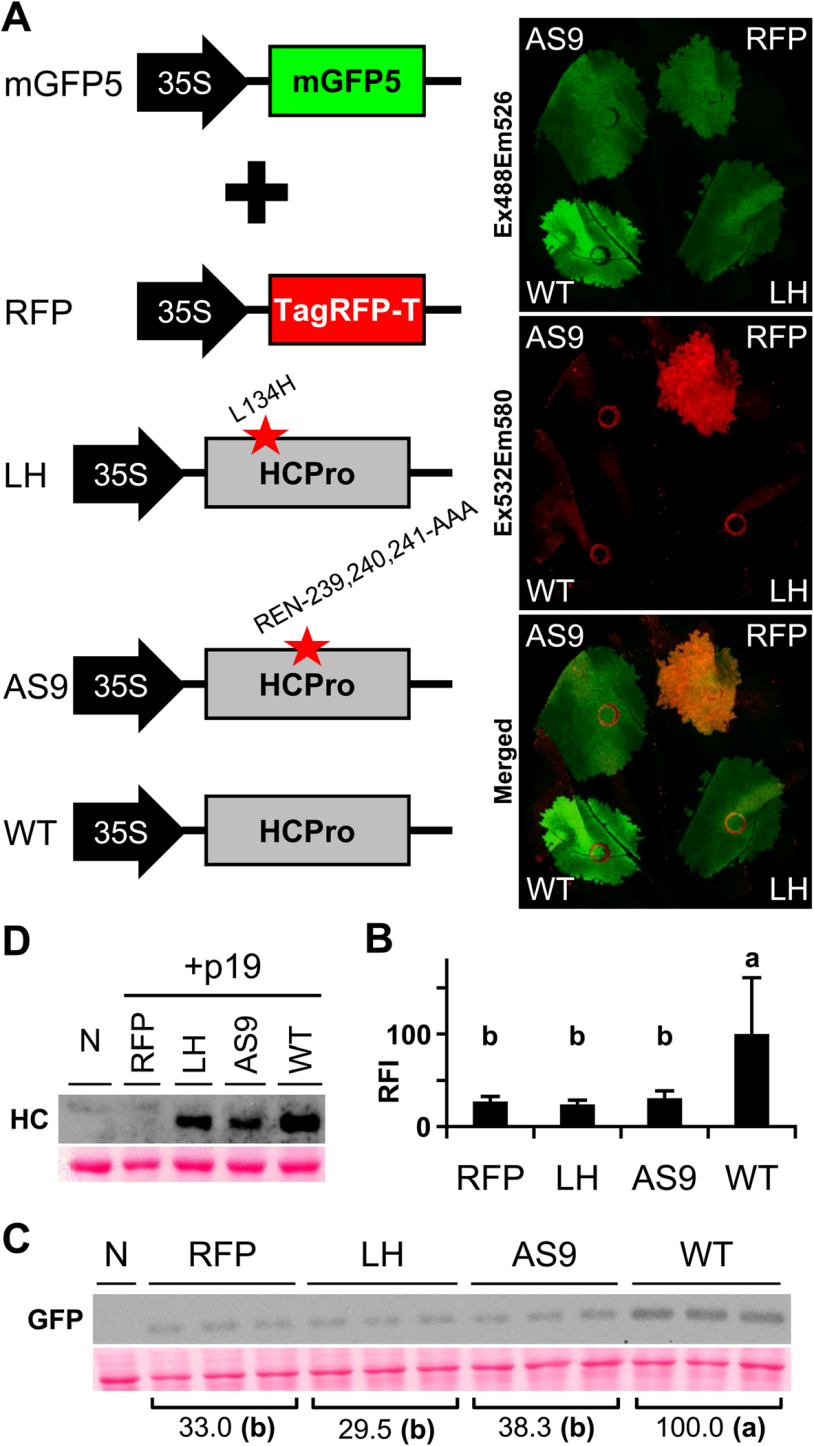
**Quantification of GFP accumulation in transient RNA silencing assay. (A)** GFP was transiently expressed by co-infiltrating *N. benthamiana* leaf tissue with an *Agrobacterium* pBin-35S-mGFP5 culture plus cultures of *Agrobacterium* containing pSN.5 TagRFP-T (RFP), pSN.5 HC-L134H (LH, producing PPV HCPro L134H mutant), pSN.5 HC-AS9 (AS9, producing a PPV HCPro mutant in which amino acids REN-239, 240, 241 were replaced by alanines) or pSN.5 wtHC (WT, producing wild-type PPV HCPro). At 6 dpa, leaf fluorescence was acquired by laser scanning using Ex488/Em526 (green) and Ex532/Em580 (red); the image overlay is shown (Merged). **(B)** GFP fluorescence intensity of the agro-infiltrated leaf patches was quantified in a 96-well plate reader. RFI was plotted using WT mean value equal to 100. Bar graph shows mean ± SD (*n* = 14 biological replicates from two independent *Agrobacterium* cultures); the difference between the results marked with different letters is statistically significant, *p* < 0.01, one-way Anova and Tukey’s HSD test. **(C)** GFP protein accumulation in infiltrated leaves at 6 dpa was assessed by immunoblot analysis. Relative GFP signal intensities are indicated using average WT equal to 100; the difference between the values marked with different letters is statistically significant, *p* < 0.01, one-way Anova and Tukey’s HSD test. Each lane represents a pool of 3 or 4 leaf samples infiltrated with two independent *Agrobacterium* cultures. N, non-treated leaf sample. Ponceau red-stained blot as loading control. **(D)** HCPro expression by the binary vectors tested was assessed by HCPro immunoblot analysis of leaf co-infiltrated with an *Agrobacterium* pSN.5 p19 culture (6 dpa). Each lane represents a pool of infiltrated leaf samples. N, non-treated leaf sample. Ponceau red-stained blot as loading control.

To determine whether leaf disc fluorescence intensity can be used for quantitative analysis of GFP accumulation in leaf tissue, we co-expressed mGFP5 with PPV silencing suppressor constructs. These included HCPro with the parent sequence (WT), with the L134H substitution (LH; which abolishes RNA silencing suppression activity [40,41]), and HCPro into which amino acids REN-239, 240, 241 were replaced by alanines (AS9). The AS9 construct was tested since the corresponding HCPro mutants in *Tobacco etch virus* (TEV) and *Turnip mosaic virus* (TuMV) are silencing suppression-defective [42-44], but no data are available for PPV. The red TagRFP-T was also included to test for interference with mGFP5 fluorescence analysis (Figure 3A). At 6 dpa, laser scanner imaging detected bright fluorescence in patches in which mGFP5 was co-delivered with wild-type HCPro (WT, Figure 3A). Analysis on a 96-well plate reader showed a significantly higher fluorescence signal in WT samples than in those of the other constructs tested, i.e., LH, AS9 and red fluorescent protein samples (Figure 3B). Fluorescence intensity in AS9 samples was equivalent to that in silencing suppression mutant L134H samples. These results suggest that the PPV HCPro AS9 (REN-239, 240, 241 replacement) construct behaves like the TEV and TuMV HCPro AS9 mutants. In immunoblot analysis, mGFP5 protein accumulation correlated positively with fluorescence signal quantification values (Pearson R^2^ = 0.9989; Figure 3C). In a parallel experiment, transient delivery of HCPro proteins was confirmed by anti-PPV HCPro immunoblot analysis of samples co-infiltrated with p19 (Figure 3D). We detected no TagRFP-T interference in mGFP5 quantification assays (Figure 3A, B).

### Monitoring of plant viral amplification dynamics by fluorometer analysis

**Figure 4:**
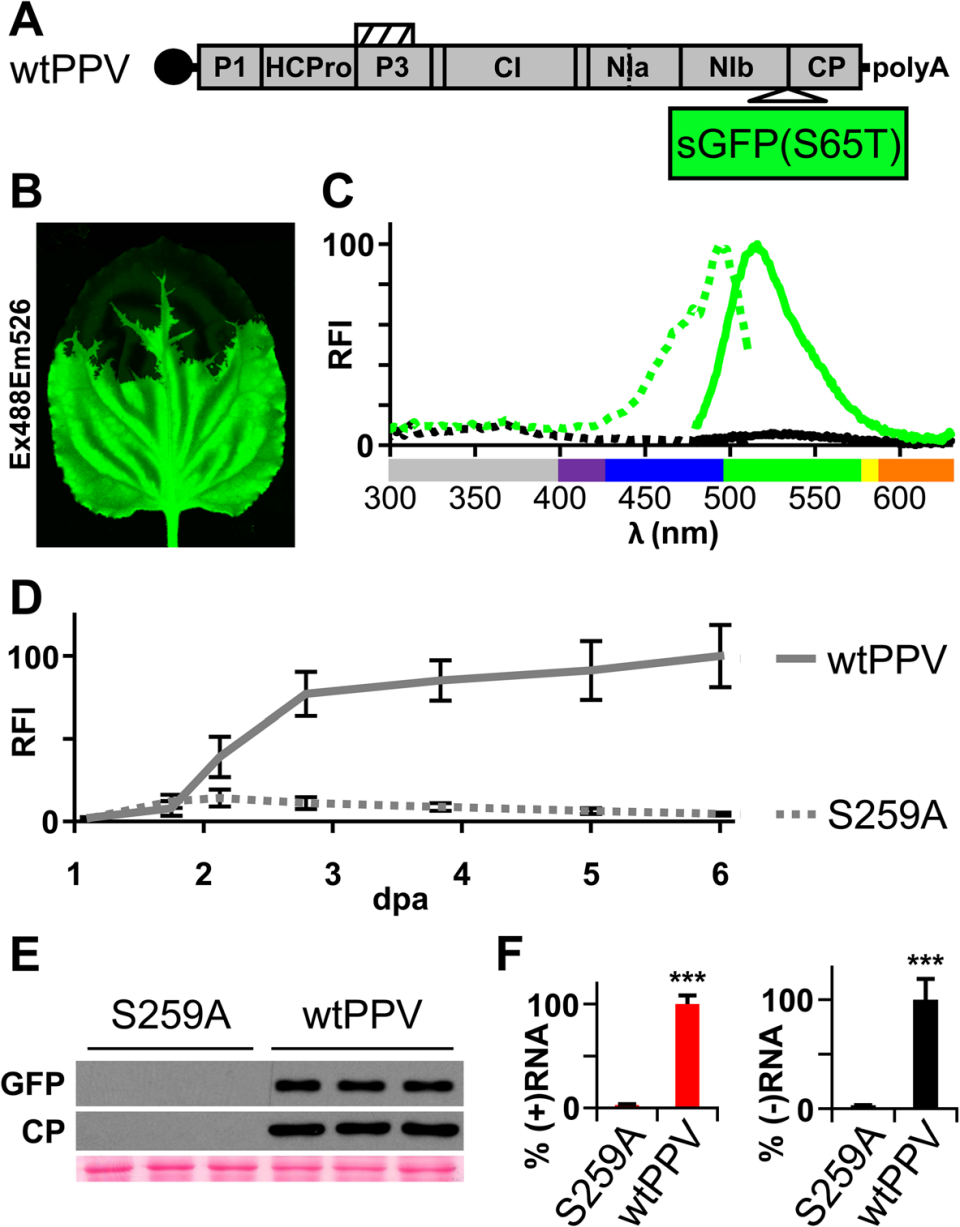
**Monitoring of GFP-tagged virus amplification dynamics by fluorescence spectroscopy.** GFP-tagged viral cDNA clones pSN-PPV (wtPPV, wild-type PPV) and pSN-PPV P1-S (S259A, in which P1 protease catalytic amino acid S259 was replaced by alanine) were delivered to plants by agro-infiltration. **(A)** Diagram of wild-type PPV (wtPPV) genome originated following pSN-PPV agro-infiltration. Hatched box indicates P3N-PIPO protein. The reporter sGFP(S65T) gene is inserted between NIb and CP coding sequences. **(B) ***N. benthamiana* plants were challenged with pSN-PPV, and fluorescence of systemically infected leaves was detected by laser scanning (10 dpa; green). **(C)** Excitation (dotted lines) and emission spectra (solid lines) of sGFP(S65T) were measured from pSN-PPV agro-inoculated leaf discs (green); leaves infiltrated with an *Agrobacterium* culture without expression vectors were used as control (black). Relative fluorescence intensity (RFI) was plotted using sGFP(S65T) peaks equal to 100. UV wavelengths are in gray, visible spectrum colors were assigned as described [39]. **(D)** GFP fluorescent intensity (RFI) from infiltrated leaves was quantified in a 96-well plate reader and plotted using average wtPPV value at 6 dpa equal to 100. Line graph shows mean ± SD (*n* = 16 samples/condition, from two independent *Agrobacterium* cultures). **(E)** Amount of GFP protein and PPV CP in infiltrated leaves at 6 dpa was assessed by immunoblot analysis. Each lane represents a pool of 3 or 4 leaf samples infiltrated with two independent *Agrobacterium* cultures. Ponceau red-stained blot is shown as loading control. **(F)** Amount of viral (+)RNA and (−)RNA from inoculated leaves at 6 dpa was quantified by RT-qPCR and plotted using average wtPPV value equal to 100. Bar graph shows mean ± SD (*n* = 4 biological replicates, from two independent *Agrobacterium* cultures); ***p < 0.001, Student’s *t*-test.

We used sGFP(S65T), a synthetic GFP version with enhanced brightness [30], as a sensitive reporter to follow PPV early amplification in plant tissue. The pSN-PPV binary vector [45] was used to deliver sGFP(S65T)-tagged PPV by agro-inoculation (Figure 4A). As anticipated, sGFP(S65T) fluorescence was readily detected in infected leaves (Figure 4B). Fluorophore spectra were confirmed by analysis of leaf discs from inoculated leaves. Compared to mGFP5, sGFP(S65T) retained the blue light excitation peak but lacked the UV peak (Figure 4C).

We further compared GFP fluorescence intensity (FI) signal dynamics of leaves agro-inoculated with pSN-PPV (wtPPV) or with pSN-PPV P1-S (S259A), a cDNA clone of a non-infectious PPV mutant with silencing suppression defects [45]. Whereas the FI of the PPV S259A clone peaked at 2 dpa, FI of wtPPV continued to increase over the 6-day time course (Figure 4D). In agro-inoculated leaves, fluorescence quantification results were corroborated by immunoblot analysis of GFP and PPV coat protein (CP, Figure 4E). We developed a strand-specific quantification of PPV RNA by RT-qPCR assay (Additional file 1), and viral RNA amounts at 6 dpa were consistent with protein determinations (Figure 4F). β-glucuronidase and luciferase genes can be used to analyze potyviral accumulation, genome amplification rates and cell-to-cell movement [14,46-48]; here we show that detection of a GFP-tagged virus is quite straightforward, since no substrates/co-factors are needed and sample preparation requirements are minimal.

### Direct leaf disc analysis of engineered monomeric fluorescent proteins

**Figure 5:**
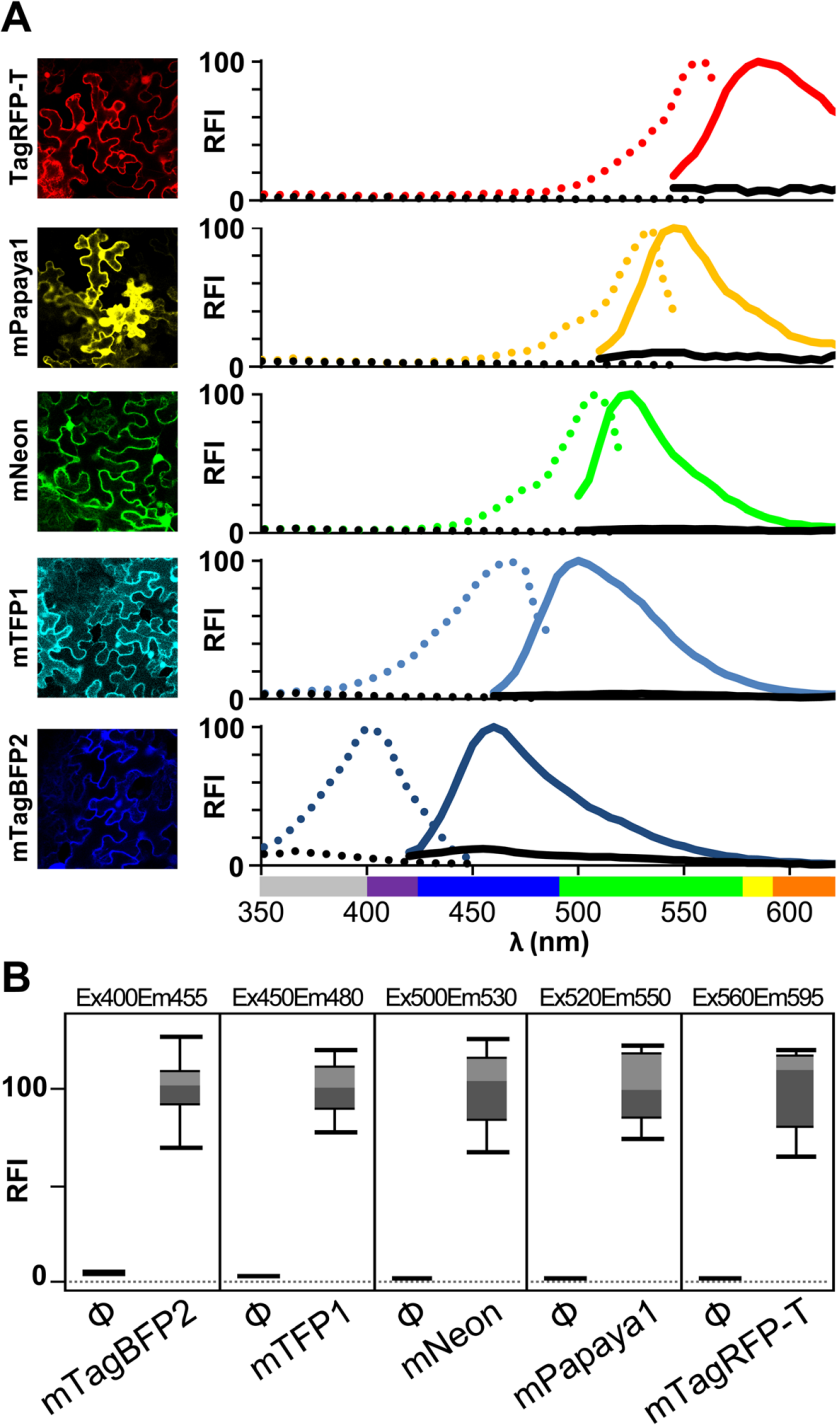
**Direct leaf disc analysis of engineered monomeric fluorescent proteins.** Fluorescent proteins were transiently expressed by co-infiltrating *N. benthamiana* leaves with an *Agrobacterium* pSN.5 p19 culture plus cultures of *Agrobacterium* containing pSN.5 mTagBFP2 (mTagBFP2), pSN.5 mTFP1 (mTFP1), pSN.5 mNeon (mNeon), pSN.5 mPapaya1 (mPapaya1), pSN.5 TagRFP-T (TagRFP-T) or a strain with no expression vector (Φ). **(A)** At 6 dpa, cell fluorescence was imaged by confocal microscopy. Fluorophore excitation (dotted lines) and emission spectra (solid lines) from agro-infiltrated leaf discs were measured in a 96-well plate reader. Leaves infiltrated with an *Agrobacterium* culture without expression vectors were used as control (black lines). Relative fluorescence intensity (RFI) was plotted using fluorophore peaks equal to 100. UV wavelengths are in gray, visible spectrum colors were assigned as described [39]. **(B)** Box-plot graphs show quantification values from *n* = 8 samples/condition. Fluorescence intensity of the leaf discs agro-infiltrated with the indicated fluorescent protein-expressing plasmid or without expression vector (Φ) was measured in a monochromator-based plate reader. RFI was plotted using each fluorophore mean value equal to 100.

There is a wide variety of engineered fluorescent proteins with improved optical and stability properties and many spectral variants were obtained by evolution of the *A. victoria* GFP sequence. For multicolor experiments, however, fluorescent proteins with minimal sequence similarity are desirable, to reduce post-transcriptional gene silencing events and assure immunodetection specificity. We evaluated the novel bright fluorescent proteins blue mTagBFP2 [31], cyan mTFP1 [32], green mNeonGreen [33], yellow mPapaya1 [34] and red TagRFP-T [35], all derived from species other than *A. victoria* (Table 1), for transient expression in plants. Fluorophore spectral properties and fluorescence intensity were easily determined using intact leaf discs collected from tissue agro-infiltrated with the corresponding constructs (Figure 5). We also show that the FI of different fluorophores can be measured simultaneously and, in multicolor experiments, the choice of reporters with minimal spectral overlap assures signal specificity (Additional file 2).

## Conclusions

We present laser scanning as an alternative method for fluorescence imaging of plant samples that, due to their size, cannot be acquired in their entirety in standard fluorescence stereomicroscopes. Dual-color fluorescence imaging of leaf samples is achieved using fluorophore combinations with minimal spectral overlap, such as mGFP5/TagRFP-T and mTFP1/mPapaya1, and image analysis can be used for raw quantitative comparisons.

We show that fluorescence plate readers are extremely powerful tools for medium-high throughput analysis of fluorescent proteins expressed in plant tissue, making it feasible to collect data from a 96-well plate in a few minutes. Fluorescence intensity is readily quantified in leaf discs, with no need to prepare protein extracts. A large number of improved fluorescent proteins have been developed, and proteins with reduced biological half-life, rapid choromophore maturation and photoactivable variants [3,49-51] might be used to increase assay sensitivity and temporal resolution for kinetic studies. We show that co-expression of TagRFP-T has no appreciable effect on fluorescence intensity quantification of mGFP5. A battery of fluorescent proteins that have minimal sequence identity with the widely used *A. victoria* GFP sequence was quantified easily in a monochromator-type plate reader. We anticipate that the method presented will aid in the design of fluorescence-based experiments with single and multiple reporter genes and facilitate comparisons of fluorophore amounts.

## Methods

### DNA plasmids and constructs

The binary vector pSN-PPV bearing a full-length cDNA copy of a PPV isolate and its variant pSN-PPV P1-S were reported [45]. An *Agrobacterium* strain GV3101 containing the binary vector pBin-35S-mGFP5 was kindly provided by D. Baulcombe (University of Cambridge, Cambridge, UK). For the remaining transient expression vectors, genes of interest were inserted into XbaI/PmlI-digested pSN2-ccdB [45] by Gibson assembly [52]. Briefly, to obtain pSN.5 TagRFP-T (encoding a mutant red TagRFP), the TagRFP sequence was amplified from pSITEII-6C1 [53] and the S158T mutation [35] was inserted by the overlap extension method [54]. For pSN.5 mTagBFP2, blue mTagBFP sequence was amplified from pGGC024 [55] (kindly provided by J. Forner, Universität Heidelberg, Heidelberg, Germany), and the I174A mutation [31] was inserted. For pSN.5 mTFP1, the cyan mTFP1 sequence was synthesized *de novo* (GeneArt, Life Technologies). For pSN.5 mNeon, green the mNeonGreen sequence was amplified from pICSL80019 [56], kindly provided by M. Youles (The Sainsbury Laboratory, Norwich, UK). For pSN.5 mPapaya1, the yellow mPapaya1 sequence was synthesized *de novo* (GeneArt, Life Technologies). For pSN.5 wtHC, PPV HCPro was amplified from pSN-PPV ∆P1 [45]; for pSN.5 HC-L134H, PPV HCPro was amplified from pSN-PPV ∆P1 and the L134H mutation inserted, whereas for pSN.5 HC-AS9, PPV HCPro was amplified from pSN-PPV ∆P1 and amino acids REN-239,240,241 were replaced by alanines. For pSN.5 p19, tomato bushy stunt virus p19 was amplified from pBIN61-P19 [38]. In all the newly-generated constructs, coding sequences are driven by a double enhancer *Cauliflower mosaic virus* 35S promoter, flanked by PPV 5’UTR and 3’UTR, followed by a nopaline synthase terminator.

### Plant agro-infiltration

*Nicotiana benthamiana* and *N. clevelandii* were grown in a greenhouse maintained at a 16 h light/8 h dark photoperiod, temperature range 19-23°C. Agro-infiltration of *N. benthamiana* and *N. clevelandii* plants was as described [6]; whenever possible, tested constructs were delivered in individual patches of the same leaf. The viral replication assay was conducted in three-week-old *N. clevelandii* plants following agroinfiltration and sampling guidelines [14], with the exception that a saturating concentration of *Agrobacterium* (OD_600_ 1.0) was used.

### Laser scanner imaging

Plant leaves were sandwiched between two low-fluorescence glasse plates and fluorescence was acquired in a laser scanner (Typhoon 9400, GE Healthcare). Settings used were normal sensitivity, focal plane +3 mm and 50–100 μm pixel resolution; excitation lasers and emission filters used are summarized in Table 1. Signal saturation was avoided by adjusting photomultiplier tube voltage. Typhoon data were exported to 16-bit .tiff files. ImageJ software [57] was used to produce false-color images and overlays, and to generate 3D-projections through the Interactive 3D Surface Plot plug-in.

### Fluorescence intensity measurements

Black 96-well flat-bottom plates (Nunc) with 50 μL water/well (to limit sample dehydration) were used for the assay. A cork borer was used for tissue sampling; individual 5.0 mm-diameter leaf discs, collected at the same distance from the infiltration point, were placed upside down in the prepared plates. Top reading measurements were used to acquire fluorescent protein excitation, emission spectra and intensity quantification in a monochromator-based plate reader (Infinite M200, Tecan Group). Gain value was adjusted manually to avoid signal saturation. RFI was quantified using the excitation and emission bands indicated in Table 1. Top reading GFP fluorescence intensity was alternatively quantified in an Appliskan (Thermo Fisher Scientific) and/or Victor X2 (PerkinElmer) filter-based plate readers.

### Western blot assays

Liquid nitrogen-frozen plant tissue was homogenized in a TissueLyzer bead mill (Qiagen). Total proteins were extracted, separated by glycine-SDS-PAGE and electroblotted onto a nitrocellulose membrane, as reported [45]. Proteins were detected using rabbit anti-PPV CP and -PPV HCPro sera, and mouse anti-GFP monoclonal antibody (clones 7.1 and 13.1, Roche) as primary antibodies; horseradish peroxidase-conjugated goat anti-rabbit IgG (Jackson) or sheep anti-mouse IgG (GE Healthcare) were used as secondary antibody. For signal quantification, chemiluminescence was acquired in a ChemiDoc XRS imager (BioRad) and analyzed with ImageJ.

### RT-qPCR

Total RNA was extracted with the FavorPrep Plant Total RNA Mini kit (Favorgen), including on-column DNAseI treatment. Purified RNA was quantified spectrophotometrically by NanoDrop (Thermo Fisher Scientific) and concentration adjusted to 50 ng/μL. Strand-specific cDNA for PPV RNA was synthesized for at least three biological replicates per condition using tagged cDNA primers in the RT step [58]. The 10-μL RT reactions contained 100 ng of total RNA and (at final concentrations) 1x Superscript III first-strand buffer, 0.5 mM of each dNTP, 5.0 mM dithiothreitol, 1.0 U/μL RiboLock (Fermentas), 5.0 U/μL Superscript III (Invitrogen) and 50 nm primer Q26_R or Q29_F (Additional file 1) to transcribe cDNA from positive and negative PPV genomes, respectively. Mixtures were incubated (35 min at 56°C, 10 min at 95°C), cooled to room temperature and diluted 1/10 - 1/25 with nuclease-free water. Technical triplicate 8 μL qPCR reactions were prepared in 384-well optical plates using 4 μL diluted cDNA sample, 1x Hot FIREPol EvaGreen qPCR Mix Plus (Solis BioDyne), 195 nM each of primer pair Q27_F/Q28_R, or 300 nM each of primer pair Q30_F/Q31_R (Additional file 1) for quantification of positive and negative PPV genomes, respectively. In a 7900HT Fast Real-Time PCR System (Applied Biosystems), reactions were subjected to 10 min at 95°C activation step, 40 cycles of 95°C, 30 s and 60°C, 60 s, followed by a final dissociation curve analysis step. Absolute quantification was done using external DNA standard curves [59]. Briefly, *Nicotiana* plants were agro-inoculated with pSN-PPV, total RNA was purified from systemically infected tissue and reverse-transcribed using the High-Capacity cDNA Archive Kit (Applied Biosystems). cDNA was used as template for PCR reactions which contained primer pair Q25_F/Q26_R or primer pair Q23_R/Q29_F for positive and negative PPV genomes, respectively. Amplicons were gel-purified and serially diluted to generate qPCR standard templates. Strand specificity of RT-qPCR assays was evaluated using synthetic positive and negative strand PPV RNA fragments. The T7 Φ2.5 promoter sequence was incorporated into PCR fragments amplified using primers Q22_F/Q23_R and Q24_R/Q25_F for positive and negative RNA strand templates, respectively. *In vitro* transcription and RNA purification were as described [45]. Healthy *Nicotiana* total RNA was used as carrier for 10-fold dilutions of target RNA alone or with a fixed amount of complementary RNA. RNA samples were reverse-transcribed in triplicate and used as template in qPCR reactions, as above.

## Abbreviations

GFP: Green fluorescent protein; PPV: *Plum pox virus*; UV: Ultraviolet; (R)FI: (Relative) Fluorescence intensity; dpa: Days post-agro-infiltration; CP: Coat protein.

## Competing interests

The authors declare that they have no financial or other competing interests.

## Authors’ contributions

Conceived and designed the experiments: FP, SK, PN, CS-M, JAG. Performed the experiments: FP. Analyzed the data: FP, JAG. Contributed reagents/materials/analysis tools: FP, CS-M, JAG. Wrote the paper: FP, JAG. All authors read and approved the final manuscript.

## Supplementary Material

Additional file 1 **Primers and PPV target region used in RT-qPCR viral RNA quantification.** (A) Sequence and use of the RT-qPCR primers. Nucleotides identical to pSN-PPV-derived viral RNA sequence are shown in uppercase letters. Non-viral tag sequences are in bold, 5' clamps to increase annealing stability are underlined and the T7 Φ2.5 promoter sequence is double-underlined. Application as follows: T7(+), *in vitro* transcription of positive strand RNA with T7 RNA polymerase; T7(−), *in vitro* transcription of negative strand RNA with T7 RNA polymerase; S(+), generation of template for positive strand standard curve; RT(+), positive strand-specific cDNA synthesis; Q(+), qPCR amplification of positive strand; S(−), generation of template for negative strand standard curve; RT(−),negative strand-specific cDNA synthesis; Q(−), qPCR amplification of negative strand. (B) Detailed scheme of the pSN-PPV binary vector used for PPV delivery to plants. P3N-PIPO protein was omitted for clarity. A 189 bp intron from the potato ST-LS-1 gene, inserted in the P3 sequence [GenBank:EF569215.1] to increase cDNA vector stability [60], is shown as a hatched box. Region flanking the P3 splicing site of pSN-PPV-derived viral RNA is shown. Positive (+) and negative (−) PPV sequences are represented with the primers used for RT-qPCR quantifications. Reverse transcription primers were designed to span the P3 intron junction of spliced viral RNAs. Brackets indicate qPCR amplicon regions. Diagram is not to scale. (C) Strand specificity of RT-qPCR assays. Standard curves were generated from cDNA synthesis reactions into which target RNA was mixed with 100 ng of *Nicotiana* total RNA alone (circles) or in the presence of a competing strand (squares). Cycle threshold numbers were plotted against the logarithm of target RNA. Click here for file

Additional file 2 **Quantification of engineered monomeric fluorescent proteins in multicolor experiments.** Fluorescent proteins were transiently expressed by co-infiltrating *N. benthamiana* leaves with an *Agrobacterium* pSN.5 p19 culture plus cultures of *Agrobacterium* containing pSN.5 mTagBFP2 (mTagBFP2), pSN.5 mTFP1 (mTFP1), pSN.5 mNeon (mNeon), pSN.5 mPapaya1 (mPapaya1) or pSN.5 TagRFP-T (TagRFP-T). At 6 dpa, fluorescence intensity of the leaf discs agro-infiltrated with the indicated fluorescent protein-expressing plasmid was measured in a monochromator-based plate reader. Evaluated excitation and emission wavelengths are shown on the left, and summarized in Table 1. Box-plot graphs show quantification values from *n* = 8 samples/condition. FI is expressed in arbitrary units. Click here for file
