# Supplementary figures and images for: Rapid fluorescent reporter quantification by leaf disc analysis and its application in plant-virus studies
Source: Plant Methods. 2014 Jul 5;10:22. doi: 10.1186/1746-4811-10-22 (PMC4105834; doi:10.1186/1746-4811-10-22)

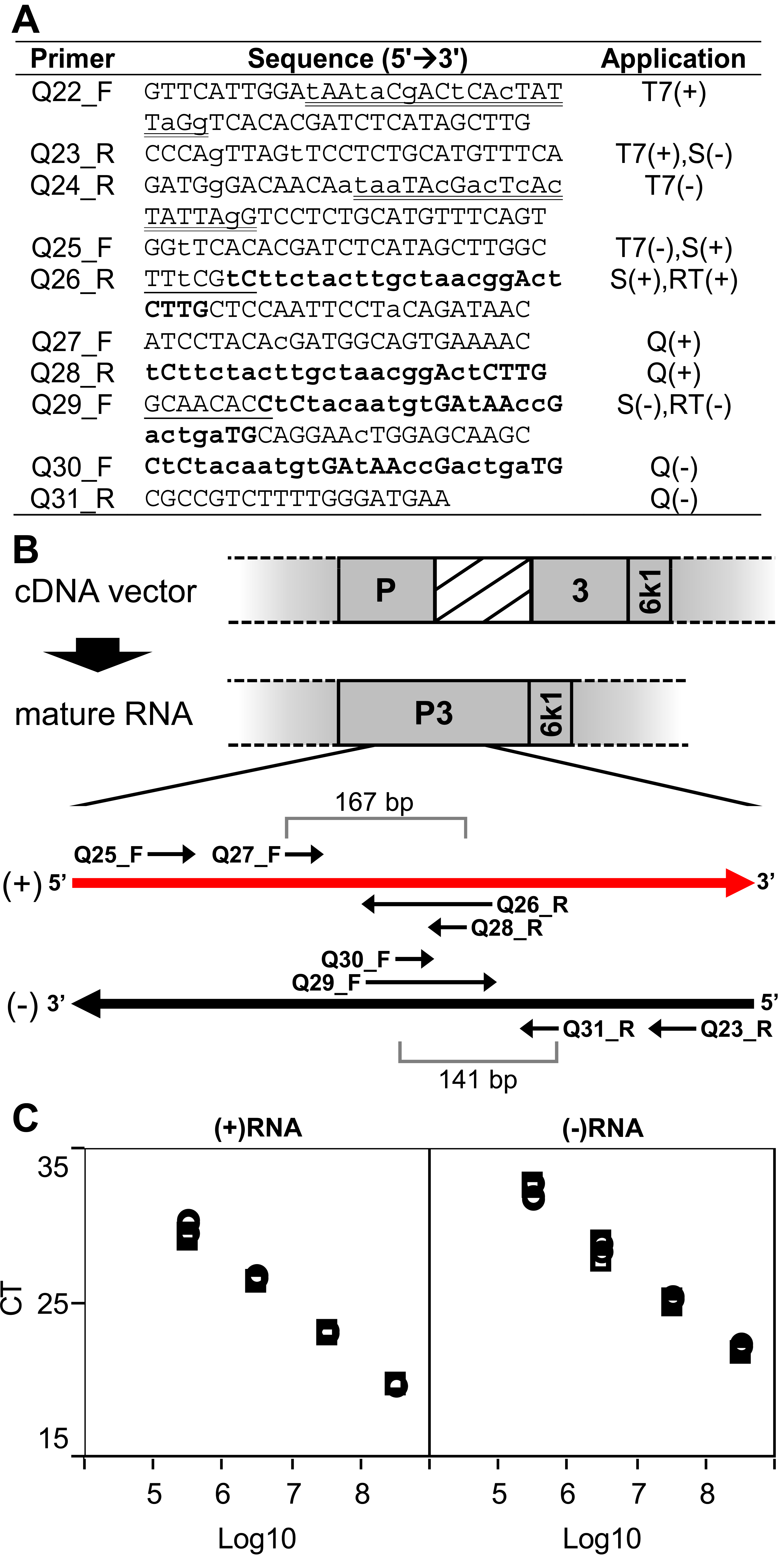

Supplement: Additional file 1 — Primers and PPV target region used in RT-qPCR viral RNA quantification. (A) Sequence and use of the RT-qPCR primers. Nucleotides identical to pSN-PPV-derived viral RNA sequence are shown in uppercase letters. Non-viral tag sequences are in bold, 5' clamps to increase annealing stability are underlined and the T7 Φ2.5 promoter sequence is double-underlined. Application as follows: T7(+), in vitro transcription of positive strand RNA with T7 RNA polymerase; T7(−), in vitro transcription of negative strand RNA with T7 RNA polymerase; S(+), generation of template for positive strand standard curve; RT(+), positive strand-specific cDNA synthesis; Q(+), qPCR amplification of positive strand; S(−), generation of template for negative strand standard curve; RT(−),negative strand-specific cDNA synthesis; Q(−), qPCR amplification of negative strand. (B) Detailed scheme of the pSN-PPV binary vector used for PPV delivery to plants. P3N-PIPO protein was omitted for clarity. A 189 bp intron from the potato ST-LS-1 gene, inserted in the P3 sequence [GenBank:EF569215.1] to increase cDNA vector stability [60], is shown as a hatched box. Region flanking the P3 splicing site of pSN-PPV-derived viral RNA is shown. Positive (+) and negative (−) PPV sequences are represented with the primers used for RT-qPCR quantifications. Reverse transcription primers were designed to span the P3 intron junction of spliced viral RNAs. Brackets indicate qPCR amplicon regions. Diagram is not to scale. (C) Strand specificity of RT-qPCR assays. Standard curves were generated from cDNA synthesis reactions into which target RNA was mixed with 100 ng of Nicotiana total RNA alone (circles) or in the presence of a competing strand (squares). Cycle threshold numbers were plotted against the logarithm of target RNA. [file 1746-4811-10-22-S1.tiff]

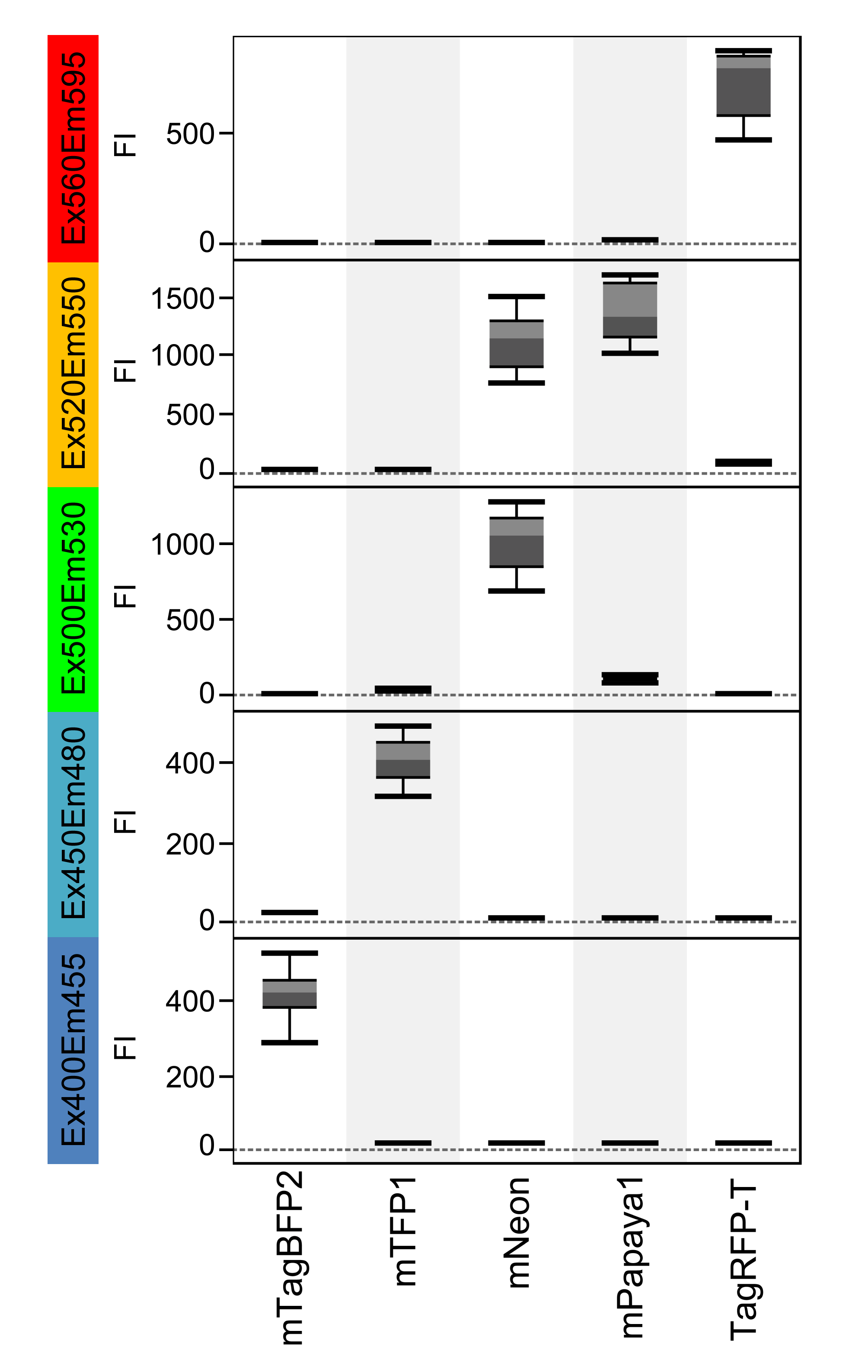

Supplement: Additional file 2 — Quantification of engineered monomeric fluorescent proteins in multicolor experiments. Fluorescent proteins were transiently expressed by co-infiltrating N. benthamiana leaves with an Agrobacterium pSN.5 p19 culture plus cultures of Agrobacterium containing pSN.5 mTagBFP2 (mTagBFP2), pSN.5 mTFP1 (mTFP1), pSN.5 mNeon (mNeon), pSN.5 mPapaya1 (mPapaya1) or pSN.5 TagRFP-T (TagRFP-T). At 6 dpa, fluorescence intensity of the leaf discs agro-infiltrated with the indicated fluorescent protein-expressing plasmid was measured in a monochromator-based plate reader. Evaluated excitation and emission wavelengths are shown on the left, and summarized in Table 1. Box-plot graphs show quantification values from n = 8 samples/condition. FI is expressed in arbitrary units. [file 1746-4811-10-22-S2.tiff]
